# Regionalization of housing policies? An exploratory study of Andalusia, Catalonia and the Basque Country

**DOI:** 10.1007/s10901-016-9528-z

**Published:** 2016-10-01

**Authors:** Kees Dol, Estrella Cruz Mazo, Núria Lambea Llop, Joris Hoekstra, Gala Cano Fuentes, Aitziber Etxezarreta Etxarri

**Affiliations:** 1 Faculty of Architecture and the Built Environment, Delft University of Technology, Julianalaan 134, 2628 BL Delft, The Netherlands; 2 University of Seville, Seville, Spain; 3 University Rovira i Virgili, Tarragona, Spain; 4 Delft University of Technology, Delft, The Netherlands; 5 University of Murcia, Murcia, Spain; 6 University of the Basque Country, Leioa, Spain

**Keywords:** Spain, Crisis, Regional housing policies, Regional welfare

## Abstract

The Spanish home ownership sector has been hit hard by the economic crisis. Repossessions stand at around half a million in the period from 2008 to 2014. This article investigates how the authorities, both at the level of the Spanish state and of the autonomous communities (regions), have responded to this problem. We investigated whether they assist troubled home owners and aim to design a less risky housing system, with more (social) rental housing. Our research in Catalonia, the Basque Country and Andalusia shows that Autonomous Communities are playing an increasingly important role in this matter. This finding fits well with theories on the formation of regional varieties of welfare, which indicate that flaws of the central governments in providing social welfare, are increasingly addressed by regions. The Basque Country seems to be on the way of designing the most comprehensive system of housing policies of the three regions, including a strong Right to Housing. All three regions regard the mobilisation of the large vacant dwelling stock as an important means to provide more affordable rental housing. However, the owners are often unwilling and the three regions have proposed drastic measures, such as fines and even temporary expropriations. The central government resists such measures, because they might interfere with the proper working of the country’s financial system. It shows that certain policy competences can never be totally isolated from other policy fields and multi-level distribution of competences makes it all the more complex.

## Introduction

Spanish society has carefully nurtured a housing model based on home ownership, with about 85 % of households being owner-occupiers at the start of the Global Financial Crisis in 2008. In retrospect this high home ownership rate included too many economically vulnerable households. Over 500,000 owner-occupiers were foreclosed during the period from 2008 to 2014 (Méndez Gutiérrez et al. 2014). Although the Spanish welfare regime has traditionally offered a high degree of job security and relatively generous unemployment benefits, this only covered the so-called insiders on permanent employment contracts. ‘Outsiders’ in flexible and informal labor have traditionally had little or no welfare entitlements (see Allen et al. 2004). The years of economic prosperity before 2008 veiled this vulnerability as ‘outsiders’ experienced relatively little unemployment. Under these favorable economic conditions, financial institutions relaxed their lending criteria and many potentially vulnerable households were able to access home ownership. From 2008, the tide turned with rising unemployment and falling house prices (Cano Fuentes et al. 2013). At the start of the crisis, the Spanish welfare state hardly had any support mechanisms for troubled households, who mainly had to turn to family (and friends) for assistance (Cano Fuentes et al. 2013; Nasarre Aznar 2014) or to organizations such as the Caritas and the Red Cross. However, the magnitude of the repossession problem has put much pressure on such support mechanisms. Another matter of serious concern is the government debt crisis. Spanish state debt exploded from one of the lowest in the Euro area in 2008 (36 % of GDP) to almost 100 % of GDP in 2015. Both the Spanish authorities and the EMU partners have called for budget cuts and fundamental welfare reform. This also involves the labor market, where measures have been taken to reduce employment protection and steer towards more flexible labor relations. Although flexibilization is commonly regarded as a way of increasing the number of jobs (see OECD 2013), the reforms may lead to an increase of households that are vulnerable to (temporary) income declines, next to the already existing population of ‘outsiders’.

Drawing on the literature it is possible to identify three main factors that contribute to the risk that emanates from the Spanish housing system (see for example Cano Fuentes et al. 2013). The first factor is the high percentage of owner occupation in Spain, which includes households that have limited financial backbone to cope with adverse economic circumstances. Affordable (social) rental housing forms a less risky alternative, but this sector makes up less than 5 % of the entire housing stock (Alberdi 2014). A second factor is the lack of housing cost assistance programs for ‘outsiders’ that experience serious income decline (see Alberdi 2014; Gentile 2016).^1^ The third factor is the ease at which mortgage lenders can evict vulnerable households from their homes (see De Weerdt and Garcia 2016; Cano Fuentes et al. 2013).

This article investigates how the Spanish authorities have responded when these three main flaws in the Spanish housing model became evident. Have they introduced policies that address the lack of affordable (social) rental housing and housing cost support? Have they taken measures to avoid speedy repossessions or have they granted basic assistance (housing) to households that have been evicted? This question needs to be analyzed from a multi-level framework, because Spain has an administrative structure where welfare policy competences are shared between the central government and the seventeen regional Autonomous Communities (Gallego et al. 2005).

Indeed this does complicate the research, but it also provides a possibility to dig deeper into the regional dimension of housing. Although the recent years have shown an increase of publications on housing policies in regions such as Scotland and Flanders (for example Robertson and Serpa 2014; Winters and Elsinga 2008), these studies hardly make an explicit link with the ‘regionalization of welfare’ literature. This is somewhat unfortunate, because about a decade ago, at least two milestone studies provided new understandings of the processes behind this regionalization (see McEwen and Moreno 2005; Ferrera 2005). So on the one hand we are ‘merely’ interested in the responses of Spanish authorities to the housing crisis, but on the other hand we have the objective to connect our research with the important field of regionalization of welfare.

The rest of the article is organized as follows. Section two gives an overview of Spanish housing against the background of regionalization and welfare restructuring. Section three gives a short overview of the method used. In section four we present an overview of recent housing policy responses to the risks associated with the Spanish home ownership model. As far as the regional level is concerned, we focus on policy responses in three representative Autonomous Communities: Andalusia, Catalonia and the Basque Country. In section five we draw some conclusions and propose an agenda for further research.

## Background: Spanish housing in a context of welfare restructuring and regionalization

This section presents the main characteristics of housing and welfare in Spain. It first sketches out the main backgrounds to the ‘regionalisation of welfare’ as found in the general literature. Then it addresses the regional dimension of Spanish governance, as policy competences are often, but not always, shared between the central state and the Autonomous Communities. The final part gives a description of the general Spanish housing model as it existed until 2008.

### The link between welfare restructuring and the emergence of regional welfare

The neo liberal project in many central state governments, with labour market reforms and the demise of generic employment protection, has been regarded as an increase of risks for vulnerable households (see Taylor-Gooby 2004). Some Northern European governments have responded to these risks by introducing targeted measures to assist these households (Bonoli and Natali 2011). What is more, such measures are sometimes designed by regional (or local) authorities and this forms the main thought behind theories of the regionalisation of welfare (Moreno 2011). In other words, when a central government retrenches and leaves few protection measures for vulnerable households, this can give incentives for regional authorities to respond by introducing their own policies. However, the process strongly relies on the (financial) autonomy of the regions and will be found mostly in decentralised states (federations). The literature also indicates that regionalization of welfare may be driven by a strive for more autonomy of local historical identities, such as in the Basque Country and Catalonia (Ferrera 2005; McEwen and Moreno 2005). There is little doubt that both the resistance to unpopular austerity measures by the central government and ambitions of ‘regional identity building’ may mutually enforce each other. The literature also notes that regional prosperity has as a role to play. Whereas some regions might favour a higher degree of autonomy, this will only be viable for the more affluent regions, because poorer regions might jeopardise (solidarity) transfers from richer regions. It is not unthinkable that it all culminates into a situation where richer regions develop more comprehensive welfare models, while the poorer regions have less to offer to their population. Although more affluent regions might not really need much state supported welfare, in some cases welfare development should be regarded as regional identity building.

### Regional varieties of welfare in Spain

It is necessary to give some background to the Spanish institutional context of welfare provision. The central state is responsible for the general economic framework. Labour market regulation and pensions are the exclusive domain of the central state (Gallego and Subirats 2012). It is thus not possible for Autonomous Communities to respond to the central government’s labor market reforms.

The Spanish Constitution (article 148 and 149) has a list of topics that are open to policy development by the Autonomous Communities (Ruiz Almendral 2003). Among these are Social Policy and Housing. However, many competences in the ‘list’ of the Spanish Constitution are shared between the central government and the Autonomous Communities (Ruiz Almendral 2003). Both levels can develop policies, which has led to repeated discussions at the Constitutional Court. The Constitutional Court can also be consulted in case new policies appear to interfere with the general fiscal and economic framework (Ruiz Almendral 2003).

Furthermore, it is necessary to make a few remarks about the income sources of the Autonomous Communities, because the literature indicates that regional income differences influence the possibilities to design regional policies. In short, the central state collects income tax and VAT. It returns 50 % of the revenues raised within an Autonomous Community to the respective Autonomous Community. In order to level out some of the regional income differences, there is an ‘equalisation transfer’ to the poorer regions (see Sollé Ollé 2013). The Autonomous Communities have the right to collect and keep all the so-called ‘ceded’ taxes, which include property taxes and stamp duties. The great exceptions to this common model are the Basque country and Navarra, which have a higher degree of autonomy and run their own tax system. These two Autonomous Communities retain all the revenues from income tax, VAT and ‘ceded’ taxes. The Basque Country pays a quotum (cupo) of about 6 % of all tax revenue for central state services (Sollé Ollé 2013; Ruiz Almendral 2003). According to Gallego and Subirats (2012) the ‘common’ financing model, with the redistribution, leads to similar budgets per capita in the ‘common’ regions. In contrast, the system in the Basque Country and Navarra, the most prosperous regions in Spain, results in much more regional government funds per capita (Gallego and Subirats 2012).

Has this all led to the formation of regional varieties of welfare in Spain? It must be said that studies that investigate the existence of regional welfare in Spain concentrate on differences in health care and education (See Gallego and Subirats 2012) because these are universal rights in Spain and are quite well developed (see Gallego et al. 2005, 2012). Social policies, including housing, are less well developed (Gallego et al. 2005; Hoekstra et al. 2011). Social care functions are mostly provided by the family, such as assisting relatives with unmanageable arrears, or taking care of older relatives. This is the main reason why the Spanish welfare regime is (still) described as familial (Allen et al. 2004). The strong employment protection of (male) breadwinners, i.e. the ‘insiders’ support this familial system.

Overall, the Basque Country is often regarded as a good example of a region that deviates from the standard Spanish ‘familial’ welfare model (Gallego et al. 2003; Vampa 2014). This is visible in a more active role of the government, in combination with a strong tradition of corporatism. Catalonia is mentioned as another example of a regional variety of welfare. It is quite active in policy development, but relies more on the involvement of private market parties in welfare provision. Overall, Gallego et al. (2003) refer to the Catalan model as *mercantil*-*comunitario*, while the Basque model is described as *publico*-*comunitario*. These two regions can be contrasted with Andalusia, the most populous Autonomous Community and a representative of the ‘standard’ familial Spanish welfare model (see Vampa 2014). Andalusia has a relatively weak economic position, which is evident from a much lower per capita income, high levels of unemployment and a high rate of social spending (Table 1). One indication for a more comprehensive welfare model in the Basque Country is that it has a higher degree of social spending than Catalonia, while it appears to have fewer problems in terms of unemployment figures.

**Table 1:** Basic demographic and economic indicators in the three selected Autonomous Communities and in Spain. *Source*: Spanish National Statistical Institute (INE) 2015

	Population (million) 2014	GDP per head (in €) 2014	Social spending per head (% GDP) 2012	Unemployment rate, QII 2015 (%)
Andalusia	8.4	16,884	21	31
Basque Country	2.2	29,683	16	16
Catalonia	7.5	26,996	14	19.1
Spain	46.8	22,780	17	22.4

### Housing policies in the regionalized Spanish context before 2008

In principle, the Autonomous Communities have been granted competences with regard to housing policies according to article 148.1.3 of the aforementioned ‘list’ of the Spanish Constitution (see Lambea-Llop 2016). The Autonomous Communities can implement their own housing policies, but only under the condition that it does not interfere with the general financial and fiscal framework of the central government. However, the central government has had a long standing tradition of providing subsidies for the *Vivienda de Protección Oficial (VPO)*—Officially Protected Housing, which in practice concerns (lower) middle-income social home ownership. VPO should mostly be regarded as a way of entering the home ownership market, rather than as a social policy that provides a low risk housing solution to vulnerable households. VPO has also been regarded as an economic stimulant. The subsidies are targeted at the construction of owner occupied dwellings and not at the support and management of a social (rental) sector.

VPO housing is usually sold well below the prevailing market price (see Hoekstra et al. 2009). In order to achieve this, various subsidy mechanisms have been used, such as provision of cheap municipal land and in some cases also cost price (or limited profit) construction by public development companies (see Alberdi 2014). The national VPO policy has given a basic framework (and budgets) for housing policy in the Autonomous Communities. Individual housing policies by the Autonomous Communities were mostly additions and modifications of the national VPO policy. Such has led to some variations between the Autonomous Communities (see Hoekstra et al. 2009).

Just about two percent of the housing stock is (social) public rental, reflecting the neglect of the sector (Alberdi 2014). In the past, central government implemented measures to give private rental tenants security of tenure and protection against rent increases, but this resulted in a retreat by private investors (Alberdi and Levenfeld 1996). Low income households have never received much government support from Spanish housing policies. There has traditionally been no system of housing benefits (Alberdi 2014). Family arrangements were important in accommodating people in precarious employment, elderly people in need of care or otherwise vulnerable family members (Table 2). This particular familial system characterizes the ‘classic’ Southern European Welfare system (see Allen et al. 2004).

**Table 2:** Tenure in Spain and in the three selected Autonomous Communities, 2011. *Source*: INE, Census 2011

	Andalusia (%)	Basque Country (%)	Catalonia (%)	Spain (%)
Home ownership				
With mortgage	34	32	34	33
No mortgage	41	46	35	39
Inherited-gift	6	6	5	7
Total home ownership	82	84	74	79
Rental	10	10	20	13
Free (or very low charge)	3	2	2	2
Other	6	4	4	5
Total	100	100	100	100

## Data and method

For this article, we consider it as too ambitious and lengthy to provide an overview of housing policy responses to the crisis in all the 17 Autonomous Communities. We rather focus on a couple of regions that reflect some of the main variations of regional welfare within Spain. Drawing on the existing literature on regional welfare as presented in the previous section, we propose to investigate the Basque Country, Catalonia and Andalusia.

Our method is straightforward. We have focused on new policy initiatives from the onset of the crisis. For this, we analyzed relevant policy documents, scientific articles and media releases. This should give us some grip on the ambitions and intentions of the Autonomous Communities. A more quantitative econometric approach is not very relevant at this point in time, because monitors and evaluations of these new policies are scarce. Also, the political context is still dynamic. A previous study on this matter found that recently introduced policies might be undone by newly elected administrations (Cano Fuentes et al. 2013).

## Responses to the housing crisis by the Spanish State and the Autonomous Communities

### Introduction

As mentioned in the introduction, the literature identifies some main policies that can help to reduce the risk of housing market booms and busts for vulnerable households. The first is to promote more affordable (social) rental housing, possibly combined with housing cost assistance for those that experience income decline. This can also cushion the effects of the expected flexibilization of the Spanish labor market. A more balanced system of repossession has also been suggested as a way to avoid the most urgent problems for individual households. Should anyone be evicted, it is helpful to have some ‘stock’ of emergency shelter housing available for those affected. It is along these lines that we investigate the responses of the central government and the three selected Autonomous Communities. At the end of this section, Table 6 gives an overview of the many measures found.

### The repossession problem

#### Policy responses: central government

In 2009, the central government offered a 2 year program with financial assistance for households at risk of eviction, the Línea ICO Moratoria Hipotecaria. It was provided under very strict conditions and assisted a total of 14,000 troubled households (ICO 2010, 2011). This program has been considered insufficient, compared to the magnitude of the arrears and repossession problems (Cano Fuentes et al. 2013). In 2012, the central government reacted by introducing a good practice code with regard to evictions in 2012 (Real Decreto-Ley 6/2012). The code proposes to restructure and negotiate debt, but the criteria are strict and form a large barrier to solutions for the majority of troubled home owners (Human Rights Watch 2014). In May 2013 the eligibility criteria were broadened, but it still rules out a majority of households (Human Rights Watch 2014). Central government also decided to temporarily suspend the eviction process for the most vulnerable by a decree in November 2012 (Real Decreto-ley 27/2012). This decree also introduced the creation of the Social Housing Fund for evicted home owners (Fondo Social de Vivienda). It includes almost 6000 vacant dwellings owned by the banks to be rented out to vulnerable households. Also here (income) requirements were strict and even after broadening the requirements not all dwellings were rented out (Human Rights Watch 2014).

Many critical Spanish commentators in a variety of media have stated that central government has had insufficient consideration for the basic housing needs of those affected by the crisis. The main complaint is that the Spanish central state is overly committed to the ‘old’ approach that includes little direct social welfare provision and that puts the market, i.e. large business interests, at center stage. The criticism cumulated in civil unrest from 2009 and onwards. The protests laid the roots for a national anti-eviction movement PAH, *the Plataforma de Afectados por la Hipoteca* (see De Weerdt and Garcia 2016; Cano Fuentes et al. 2013). It even attracted attention from renowned humanitarian organizations such as the Human Rights Watch (2014) and Amnesty International (2015). There is little doubt that the PAH brought the most poignant cases to the attention of local policy makers, who were stimulated to take action (De Weerdt and Garcia 2016).

#### Policy responses: the three Autonomous Communities

Although the impact of mortgage arrears and evictions in the Basque Country is by comparison smaller than in most other regions (Table 3), it was the first Autonomous Community to launch specific initiatives to respond to the problems. Etxezarreta et al. (2013) give an overview of the measures taken in the period from 2009 until 2013. In January 2009, the Basque government started a program to purchase dwellings of unemployed owner-occupiers (*Recompra de vivienda libre*), who could then rent the dwelling from the Basque government. The households concerned had the option of repurchasing the dwelling in a later stage, should their financial situation improve again. Although the program was aborted after a change in the Basque Government and lasted only 5 months, it shows that the Basque Country was relatively quick in taking action. Moreover, in May 2012, the Basque Country introduced a comprehensive program to avoid evictions through mortgage assessment, mediation between banks and troubled home owners, as well as offering social housing to households that had been evicted. A number of agreements, also in combination with each other, would be possible: remission of part of the debt, restructuration of the payment, entire debt cancellation, or avoiding evictions by maintaining the former owner-occupiers as (social) tenants of the mortgage lender.

**Table 3:** Foreclosures and evictions in Andalusia, Catalonia, the Basque Country and Spain as a whole. *Source*: General Council of the Judiciary (CGPJ)

	Foreclosures in process 2012	Evictions 2008–2012	Evictions per 1000 inhabitants 2008–2012
Andalusia	23,687	41,510	5.0
Catalonia	19,457	40,947	5.4
Basque Country	6750	2024	0.9
Spain	159,763	198,076	4.2

The Catalan government, in the Catalan Housing Plan of 2013–2016 (Act 75/2014) focused on measures to avoid the loss of housing. It provided subsidies to people that are at risk of eviction or that have been recently evicted. Furthermore, the Catalan Housing Agency (Ofideute), offers help and advice to people with problems to pay the mortgage (Generalitat de Catalunya 2015). They also negotiate with banks in order to avoid eviction. Recently introduced intermediate tenures in Catalonia can also be used as a means to avoid repossession: in agreement with the bank, the dwelling can be transformed into shared ownership. Furthermore, the Catalan Parliament has also committed itself to offer alternative housing (social rent) to households that are at risk of eviction. More recently, it also took the initiative (Act 24/2015) to avoid evictions by stronger measures. The authorities can force the owner to offer a 3-year social lease.

The Housing Ministry of Andalusia launched the ‘Andalusian Program in Defence of Housing’ in October 2012. This is a free public service that functions through a network of offices in the eight provinces of Andalusia with the aim of supporting citizens through prevention, mediation and protection services. In case of mortgage arrears, the program includes the possibility of mediation: the Regional Housing Ministry intermediates with financial institutions on behalf of citizens who suffer debt after losing employment, helping citizens to ensure the best possible conditions for the negotiation or settlement of the debt. Finally, the protection service establishes collaboration between the Regional Housing Ministry and the municipalities in order to provide solutions to families that have been evicted. These families must meet some general requirements (being unemployed and having little financial means) in order to be able to get a social rental dwelling. Another quite drastic initiative taken by the Andalusian government was to temporarily expropriate homes (3 years) when a bank has repossessed and threatens to evict the inhabitants (Andalusian Decree 6/2013). This measure was mainly intended to avoid social exclusion of very vulnerable households on lower incomes. The repossessed household would be able to use the dwelling but it loses the ownership of the home.

However, the Andalusian temporary expropriation measure was contested by the central government at the Constitutional Court (Judgment 93/2015). This judgment has indicated that Autonomous Communities’ housing policies cannot collide with interests of the banking sector because these are considered of crucial importance for the entire economy. Another argument is that expropriation is too radical because it restricts private property rights. In this view, the best stage to regulate these matter are at the central government level. From such a perspective, the Catalan compulsory 3 year social lease initiative (2015) for households at risk of eviction was also taken to the Constitutional Court for suspension. No decision has yet been taken.

### Provision of more affordable (social) rental housing

An increase of social housing might not only be pursued by an increase of new construction, but also by stimulating solutions in the existing vacant housing stock. This has already been touched upon in the previous section with regard to the Social Housing Fund of vacant dwellings owned by banks. In fact, uninhabited housing in Spain had become a common problem since the late 1990s, with some of the vacant dwellings being in good condition and in the right location, but not put on the market because the owners hold on to them for speculative reasons and don’t want to act as a (social) landlord (Hoekstra and Vakili-Zad 2011). This vacant dwelling problem was exacerbated during the construction boom years of 2000–2008, when much housing construction was undertaken on a speculative basis and remained unsold after the market collapsed in 2008. This resulted in a ‘stock’ of nearly 650,000 new, partly unfinished, empty dwellings after the housing market collapsed. In this section we deal with incentives for stimulating new construction as well as with the interesting theme of solutions in the existing housing stock.

#### Central government

In the context of the crisis and the numerous evictions of owner occupiers, the central government’s focus has gradually shifted towards the rental sector. The State Housing Plan 2009–2012 required that 40 % of new VPO dwellings must be realized in the rental sector (Alberdi 2014). The new 2013–2016 State Housing Plan goes much further. It lays down a public program of rental housing stock promotion and thus abolishes the old cornerstone of subsidized VPO dwellings for owner occupation (Ministerio de Fomento 2013). The new Plan includes a ‘rental pillar’ which aims to create a public or non-profit rental sector, owned by parties such as public entities, foundations or non-profit organizations.

As we will see further on, initiatives to mobilize the empty dwelling stock are mostly addressed at the level of the Autonomous Communities. The Social Housing Fund (see before) was an initiative of the central government in collaboration with the financial sector.

#### The three Autonomous Communities

The construction of new VPO-housing traditionally took place in the home ownership sector. However, from the mid-2000s, the rental sector has become more important (see Table 4). Whereas the policy of the central government has changed towards 100 % rental in the latest housing plan of 2013, there are still regions with substantial shares of home ownership dwellings within their current plans. There may be a temporal factor in this: the national plan of 2009–2012 still included 40 % rental. However, the latest Basque plan of 2013 has not shifted towards 100 % rental dwellings, but keeps a substantial share of VPO in owner occupation. It is an indication of regional divergence in housing policy: the Basque Country has a high degree of autonomy and runs its own plans.

**Table 4:** Most recent VPO housing construction plans by tenure in Andalusia, Catalonia and the Basque Country. *Sources*: Junta de Andalucia; Generalitat de Catalunya; Gobierno Vasco

	Plan Andalusia	Plan Catalonia	Plan Basque Country
	2008–2012*	2007–2016	2013–2016
Rent (%)	35	40	49
Ownership (%)	65	60	51
Total dwellings	149,600	150,000	8000

* There is no new Andalusian housing plan: only decrees have been added

**Fig. 1:**
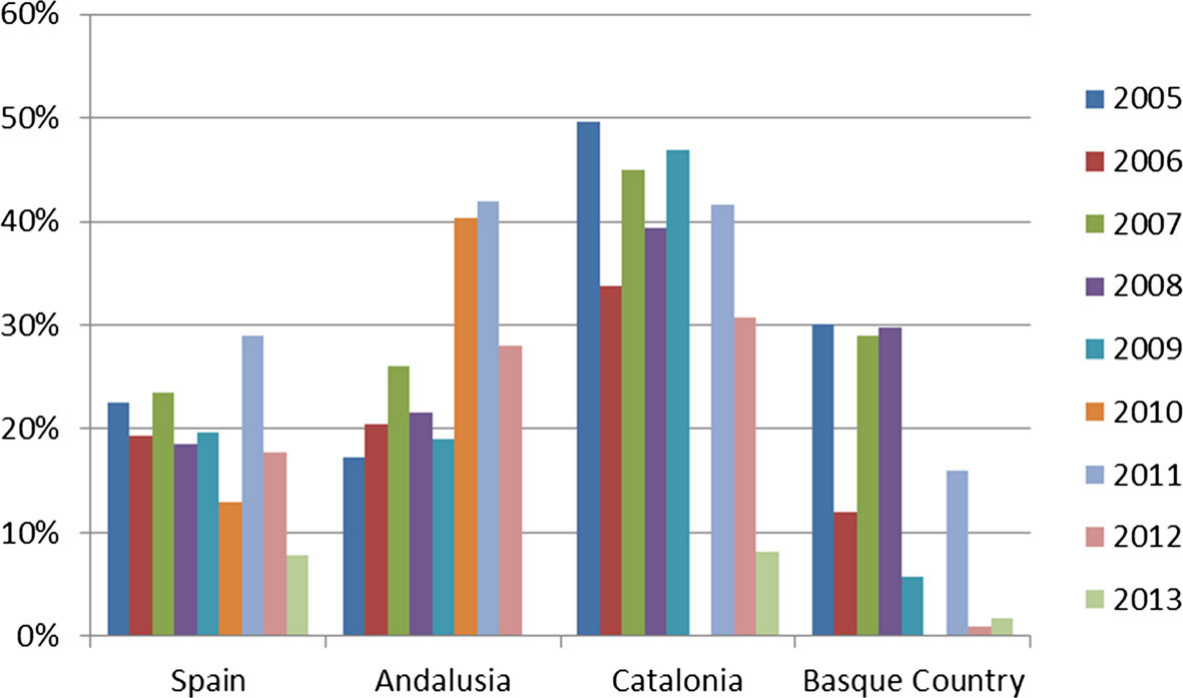
Percentage of rental housing in newly constructed VPO dwellings 2005–2013. *Source*: Ministerio de Fomento, Table 1.8, authors calculations

Statistics of newly constructed VPO dwellings show a regional variation that is more or less in line with the plans. Although Spain as a whole has shown increasing rates of rental dwellings in the VPO program before the crisis, the new policies at the central state level are entirely targeted towards the rental sector. Catalonia has had the highest rate of VPO dwellings in the rental sector of all Autonomous Communities. This is probably because of the domination of the Barcelona metropolitan area, where demand for rental housing is relatively high. Andalusia had a relatively low share of newly produced rental VPO housing until 2009, but recently it caught up. This is in line with increasing attention for the rental sector nationwide. The Basque Country has actually experienced a decline in newly produced VPO rental dwellings after 2009, but the recent Housing Plan of 2013 intends to provide half of the newly produced VPO dwellings in the rental sector (Fig. 1).

With regard to newly constructed housing, an interesting new phenomenon in Catalonia are experiments with so-called intermediate tenures (for definition and types, see Elsinga et al. 2015). Shared ownership and temporary ownership have recently been introduced in the Catalan Civil Code. Furthermore, there is a growing interest in cooperative housing as an alternative to (social) home ownership (Etxezarreta and Merino 2013). The Basque Country actively supports co-operative housing in its latest housing plan of 2013. As of today, it is not yet possible to assess whether these new initiatives have resulted in any significant rise in affordable housing production. However, they have attracted the interest of housing researchers, so in time evaluations will follow.

All three Communities experience empty housing as a problem (Table 5) and mobilization of empty housing is regarded as a means to provide more rental dwellings and alleviate pressure on the housing market.

**Table 5:** Empty newly constructed dwellings 2013. *Source*: Ministerio de Fomento (2014) and INE 2014

	Andalusia	Catalonia	Basque Country
Empty new dwellings	91,212	85,307	11,849
% of empty new dwellings of total dwelling stock (%)	2.1	2.2	1.2

In the Basque Country empty dwellings, at about 9 % of the housing stock, have long been regarded as a major issue. Already from 2003 the “Bizigune” program has attempted to place uninhabited houses in private ownership on the market for rental housing. Its’ most important features are that owners receive between 65 and 75 % of the market rental price, with the guarantee of receiving the property in similar conditions after the tenant vacates the dwelling. Tenants in the Bizigune program pay less than 30 % of their annual gross income in rent. Currently there are 5193 dwellings in this program. Since March 2012 the so-called ASAP program is in place (Decree 43/2012). In order to encourage the participation of the owners of empty homes, this program provides a system of guarantees in the form of insurance policies covering defaults, damage and legal assistance. The ASAP program target is set at around 2300 dwellings in total. Finally, the new Basque Housing Act (3/2015) puts more pressure on companies that own empty dwellings. After 2 years of vacancy, it imposes an annual fine of 10 euros per square meter. The new Act also provides the possibility of compulsory renting out of vacant dwellings. Furthermore, the Act allows the Basque authorities to temporary (3 years) expropriate dwellings that have been vacant for more than 2 years. And lastly, the new Act creates the right of first refusal for housing. In case the financial institution wishes to sell the dwelling, the Basque government has the option to buy the dwelling and rent it out as a social dwelling. This only applies to areas with high housing demand.

In Catalonia, several measures to activate the vacant dwelling stock have been introduced in the period 2008–2009. They derive from the Catalan Housing Plan 2007–2016, updated in 2012. In the Mediation Program, the Catalonian Administration acts as an intermediary, finding a tenant, ensuring an affordable rent and proper use of the dwelling, and in the Transfer Program, banks place empty dwellings under the control of the Catalonian Administration who lets them at an affordable rent. The third mechanism consists of *Avalloguer,* under which the Administration guarantees the payment of 3 months’ rent (and in special cases 6 months) should the tenant default, as long as the owner keeps the rent affordable. More drastic interventions have been proposed that compare strongly to the Basque ones. The Catalan Decree 1/2015 creates the right of first refusal for foreclosed dwellings in areas with high dwelling demand. Catalan Act 14/2015, implements a tax for landlords that keep dwellings vacant for more than 2 years, while the Catalan Act 24/2015 gives the possibility for temporary expropriation of dwellings that have been vacant for more than 2 years. It only affects property held by commercial investors and not by private households or non-profit and public entities. However, also these two measures have been brought to the Constitutional Court.

In Andalusia the numbers of empty, partly unfinished dwellings soared after massive speculative oversupply in the years before 2008. The Andalusian Decree 6/2013 on “Measures to ensure compliance with the social housing function” was introduced in order to stimulate banks to rent out their vacant dwelling portfolios. This regulation provides subsidies, while at the same time, there are annual fines up to € 9000 on dwellings that are fit for habitation, but not rented out after a vacancy of 6 months. Dwellings owned by private individuals do not fall under this regime.

On the subject of mobilisation of empty dwelling stock, problems have also arisen between the central government and the Autonomous Communities. The legal argument is complex, but it boils down to the fact that it is hard to prove the vacancy. The central government has brought the initiatives to the Constitutional Court to decide. Furthermore, temporary expropriation of empty dwellings has not gone down well with the central government, from the idea that it does not wish to devolve those competences to the regions and that it might interfere with the interests of the financial sector.

### Housing cost subsidies to vulnerable households

#### Central government

One significant new measure from the Housing Plan 2013–2016 on the national level, is the introduction of rental housing support. It is aimed at low-income families (below € 22.365 annually) and consists of an annual aid of up to 40 % of the rent, maximized at € 2400 per year (Ministerio de Fomento 2013).

Although the previous part of the article mentioned that there is no tradition of housing cost assistance in case of income decline, one measure needs to be mentioned. In 2007, the central government introduced the Renta Basica de Emancipación. It’s main aim was to assist young households up to the age of 35 to access the housing market, without having to buy a dwelling, which was the traditional pathway. It was targeted at households with an income up to € 22.000 and provided a monthly subsidy of € 210 per month for a duration of 4 years (see Gentile 2006 for a detailed description in English). In principle, this subsidy could also help young private renters that experienced a substantial income decline after becoming unemployed. However, due to the enormous debt crisis of the central government, the subsidy scheme was abolished in 2012.

#### The three Autonomous Communities

On the regional level, no such subsidies have been found. However, it is interesting to mention that two Autonomous Communities (Valencia and Madrid), regarded the central government’s Renta Basica de Emancipación as a regional competence and insisted that they establish the qualification rules, given that they can best assess local needs (see Gentile 2016).

### Right to housing

During the research process another highly relevant policy dimension was found, which concentrates on the ‘Right to Housing’. Is it possible for evicted households or inhabitants who otherwise have great problems to provide for their own housing, to legally claim housing?

Article 47 of the Spanish Constitution recognizes a ‘right to decent and adequate housing’, but the central government never implemented an explicit law (or articles) that makes this right effective. Whereas all Autonomous Communities have competences with regard to fulfilling a potential right to housing, only five Autonomous Communities have implemented legislation with a significant impact. These are Andalusia, Catalonia, the Basque Country, Aragon and Galicia. Catalonia and Andalusia have introduced Housing Acts that include a right to housing in 2007 and 2010 respectively (Tejedor Bielsa 2012). The Andalusian and Catalan legislation should not be regarded as enabling all people to legally claim a dwelling, but it is rather an attempt to provide a more concrete policy framework for the Constitution’s ‘right to decent and adequate housing’. In both Autonomous Communities, the main objective is to create legislation that regulates the distribution of (affordable) social housing to specific target groups. The Andalusian Law also prescribes an inventory of the size of the target group and the housing needed to accommodate these households.

Recently the Basque Country has approved legislation that will give the population a right to demand a dwelling (Basque Act 3/2015). In other words, it forms a hard legal right to housing, which goes beyond the Catalan and Andalusian legislation. The discussion about a right to housing in Basque Country started about 15 years ago and the process towards implementation had to overcome major concerns over practical matters, specifically whether there would be sufficient housing available in case households make legal claims based on this right to housing. The ambition is to reserve some VPO housing in order to give households the right to be re-housed in case of adverse circumstances (Olea Ferreras 2015). The Basque Parliament finally accepted the formal right to housing in June 2015 (with support of the left wing majority) and it was supposed to be effective from October 2015. However, the Spanish central government raised an objection of unconstitutionality against the entire new Basque housing Act (3/2015), because it includes aforementioned measures such as temporary expropriation and fines-taxes on unoccupied housing (Olea Ferreras 2015).

## Discussion and concluding comments

This paper explored to what extent the crisis of 2008–2014 has been a driver of changes in the Spanish housing system. A second objective was to investigate how this works out in the Spanish governance context, where regional authorities have competences to design social welfare policies, including housing. In this, the investigation offers perspectives to connect with theories of ‘the regionalization of welfare’, which has received little attention in housing research. One very relevant line of thought in the regionalization of welfare literature, is that regional authorities increasingly step into combat social risks that are not addressed by the central government, either because of past dismantling of social policies or because social policies never existed.

Surely, at both governance levels, the authorities have taken measures to create a less risky housing system. The three Autonomous Communities involved themselves in social policies to assist home owners at risk of eviction. They also aim to provide more affordable housing through the mobilization of the empty housing stock held by banks or other investors. To pursue this goal, the three regional authorities do not hesitate to take drastic measures such as temporary expropriation. Furthermore, all three Autonomous Communities have tried to give more concrete terms to the Right to Housing (article 47) in the Spanish Constitution. This should help vulnerable (evicted) households to have a roof over their head and rely less on family and NGO’s such as the Red Cross and the Caritas.

We found some regional variations in the measures taken (see Table 6), but we would take some caution in stating that they seamlessly fit into the Spanish regional welfare variations as proposed by Gallego et al. (2003). Overall, the Basque Country has committed itself most strongly to building a somewhat less risky housing system. It was quick to respond to the eviction problems and has now even designed a Housing Law which gives people a concrete claim on the Right to Housing. The Basque authorities have also committed themselves to providing emergency shelter for evicted households. The Basque policies are notable, because in relative terms the region experienced the least problems emanating from the crisis. Probably this is part of a larger process of regional identity building, which the literature regards as another important driver of regionalization of welfare (Ferrera 2005; McEwen and Moreno 2005). Overall, there may be an argument that the term *publico*-*communitario* (Gallego et al. 2003) applies to the housing policy measures taken by Basque Country. Catalonian measures compare to the Basque Country, but they do not go as far with regard to the Right to Housing. Based on the policies investigated here, we are not able to clearly confirm the existence of a distinct Catalan *mercantile*-*communitario* variety of welfare (Gallego et al. 2003) where the authorities stimulate private parties to involve themselves. With regard to the case of Andalusia we are also not able to identify a clear line. One difference between Andalusia and the other two regions is that is appears to use more sticks than carrots to mobilize the empty housing stock. Probably this is related to budgetary restrictions in this region, which has traditionally had a weaker economic backbone than Catalonia and the Basque Country. But overall, it is clear that all three communities have ambitions to play a more active and social role in housing policy. In this, we need not overlook the role of the citizens resistance movements against the evictions (Indignados, PAH), which stimulated (local) politicians to take action in those localities where initially little was done to assist troubled home owners.

**Table 6:** Responses to the eviction crisis and new policy initiatives to promote a less risky housing system (Some regional initiatives are contested by the central government: see main text)

	Assistance to troubled home owners	Construction of new affordable rental housing	Mobilisation of vacant dwellings for lower incomes	Housing cost subsidies	Right to Housing
Central government	Some financial assistance for most vulnerable households 2009–2011 Suspension of eviction for most vulnerable households Social Housing Fund (6000 units) for *evicted* households	Gradual shift from dominance of owner occupied social VPO dwellings to 100 % social rental VPO in 2013	None, although SAREB transfers of empty new dwelling complexes to investors might partly benefit lower incomes	Rental subsidy for *young* households with income < € 22.000 in private rental (2007–2012) Rental subsidy to incomes < € 22.365	Article 47 in Spanish Constitution but no specific law that makes it effective
Andalusia	Intermediation banks and owners Provide social rental for vulnerable evicted households Temporary expropriation from repossessing banks for households at risk of eviction to avoid eviction	More rental in VPO-programs	Fine of € 9.000 per vacant dwelling (2012)		Right to Housing in legislation (2010) which constitutes a framework for distribution of affordable housing to vulnerable groups. Inventories proposed to estimate size of target group
Catalonia	Ofideute consultancy-intermediation Some financial support for most vulnerable households Transformation into temporary or shared ownership Provide social rental for evicted households	More rental in VPO-programs Support for intermediate tenures	Guaranteed payments to private landlords in case of default Right of first refusal (AC has first right to buy dwelling for social purposes) Tax for dwellings, vacant > 2 years Temporary expropriation dwellings banks, vacant > 2 years		Right to Housing in legislation (2007) which constitutes a framework for distribution of affordable housing to vulnerable groups
Basque Country	Purchase of dwellings households at risk and transform into (social) rental (2009–2011) Intermediation banks and home owners Transformation into renting from the repossessing bank Provide social rental for evicted households Right to be re-housed (in specially reserved VPO housing)	More rental in VPO-programs Support for cooperatives	Lower rent in return for guaranteed payments to private landlords in case of default Right of first refusal (see Catalonia) Tax for empty dwellings > 2 years, €10/m2 (2015) Temporary expropriation dwellings banks, vacant > 2 years Mandatory lease of empty dwellings (2015)		Right to Housing in legislation (2015) which gives the population a hard claim on housing in case of homelessness (By means of specially allocated VPO housing See also bottom left)

The active role of the Autonomous Communities contrasts with the policies of the central government. Admittedly, the new rental subsidy of the central government for lower incomes is a step forward in reducing risks for vulnerable households; also in the future when employment security will be further dismantled. Another positive step is the shift towards social rental housing within the VPO programs, but it is still a small contribution to the very small social housing sector in Spain. However, the minor role of the central government in assisting foreclosed home owners has been strongly criticized and has even caught the attention of international humanitarian organizations. Under severe pressure of popular movements it took some measures to avoid the most poignant eviction cases, but it is clear that the Autonomous Communities were much more active to address the social problems. This is consistent with literature on regionalization of welfare, which indicates that regional (and local) authorities increasingly aim to tackle the flaws in central government social policy. What is more, the Autonomous Communities’ attempts to mobilize vacant dwellings through drastic measures such as temporary expropriation, have been actively blocked by the central government. It must be admitted that central government has some good legal motives to ask for suspension of such legislation, but it is a potential source of conflict. The Autonomous Communities might ask which alternatives the central government has to offer.

Overall, further research is needed. Although we focused on three important Autonomous Communities, there might be some more variation. Also, the economic circumstances in Spain are still dynamic and not all discussions between the Autonomous Communities and the central state have reached final conclusions.

Finally, we call for more regional housing research in countries with a federal structure. There is an increasing number of those studies, but they take little account of the main underlying drivers that are mentioned in the regionalization (territorialization) of welfare literature. Such studies will also be helpful for international comparative housing research, which needs to take more notice of the regional dimensions. A focus of comparative housing research to the national level might, for instance, only find patterns of government retrenchment, while there is new activity in local contexts.

## Footnotes

1 While other government provision of social benefits for ‘outsiders’ is also very limited.

## References

[CR1] Alberdi B, Scanlon W, Fernandez Arrigoitia M (2014). Social housing in Spain. Social housing in Europe.

[CR2] Alberdi B, Levenfeld G, Balchin P (1996). Spain. Housing Policy in Europe.

[CR3] Allen J, Barlow J, Leal J, Maloutas T, Padovani L (2004). Housing and welfare in Southern Europe.

[CR4] Bonoli, G., & Natali, D. (2011) *The politics of the new welfare states in Western Europe*. European University Institute Working Paper 2011/17.

[CR6] Cano Fuentes G, Etxezarreta A, Dol K, Hoekstra J (2013). From housing bubble to repossessions: Spain compared to other West European countries. Housing Studies.

[CR8] De Weerdt J, Garcia M (2016). Housing crisis: the Platform of Mortgage Victims (PAH) movement in Barcelona and innovations in governance. Journal of Housing and the Built Environment.

[CR10] Elsinga M, Hoekstra J, Dol K (2015). Financial implications of affordable home ownership products: four Dutch products in international perspective. Journal of Housing and the Built Environment.

[CR11] Etxezarreta AC, Fuentes G, Hoekstra J, Dol K (2013). Analisis multiescalar de la burbuja inmobiliaria y los desahucios: la Comunidad Autonoma de Euskadi en el contexto estatal y europeo. Revista de Estudios Regionales no.

[CR12] Etxezarreta A, Merino S (2013). Las cooperativas de vivienda como alternativa al problema de la vivienda en la actual crisis económica. REVESCO, Revista de Estudios Cooperativos.

[CR13] Ferrera M (2005). The boundaries of welfare. European integration and the new spatial politics of social protection.

[CR14] Gallego R, Goma R, Subirats J (2003). Estado de bienestar y comunidades autónomas. La decentralizacion de las políticas en España.

[CR15] Gallego R, Goma R, Subirats J, McEwen N, Moreno L (2005). Spain: From state welfare to regional welfare?. The territorial politics of welfare.

[CR16] Gallego R, Subirats J (2012). Spanish and Regional Welfare Systems: Policy Innovation and Multi-Level Governance. Regional and Federal Studies.

[CR18] Generalitat de Catalunya. (2015). *Informe sobre el sector de l’habitatge a Catalunya. Any 2014.* Generalitat de Catalunya. Departament de Territori i Sostenibilitat. Secretaria d’Habitatge i Millora Urbana.

[CR19] Gentile A (2016). Rental subsidy and the emancipation of Young adults in Spain. International Journal of Housing Policy.

[CR28] Méndez Gutiérrez del Valle R, Abad Aragón L, Plaza Tabasco J (2014). Geografía de las ejecuciones hipotecàrias en España. Collección Estudios.

[CR21] Hoekstra J, Heras Saizarbitoria I, Etxezarreta Etxarri A (2009). Recent changes in Spanish housing policies. Subsidized owner-occupancy dwellings as a new tenure sector. Journal of Housing and the Built Environment.

[CR22] Hoekstra J, Vakili-Zad C (2011). High vacancy rates and rising house prices: The Spanish paradox. Tijdschrift voor de Economische en Sociale Geografie.

[CR23] Human Rights Watch (2014). Shattered dreams: Impact of Spain’s Housing crisis on vulnerable groups.

[CR24] Instituto de Credito Oficial (2010). Informe annual 2010.

[CR25] Instituto de Credito Oficial (2011). Informe annual 2011.

[CR26] Lambea Llop N (2016). Social housing management models in Spain. Revista Catalana de Dret Public.

[CR27] McEwen N, Moreno L (2005). The territorial politics of welfare.

[CR29] Ministerio de Fomento. (2013). *Plan Estatal de fomento del alquiler de viviendas, la rehabilitación edificatoria, y la regeneración y renovación urbanas, 2013*-*2016*. Madrid, 2013.

[CR30] Ministerio de Fomento. (2014). *Informe sobre el stock de vivienda nueva 2013*. Madrid, 2013

[CR31] Moreno, L. (2011) *Multilevel citizens, new social risks and regional welfare*. Instituto de Politicas y Bienes Publicos, working paper no. 3.

[CR32] Nasarre Aznar S (2014). La vivienda en propiedad como causa y víctima de la crisis hipotecaria La vivienda en propiedad como causa y víctima de la crisis hipotecaria. Teoría y Derecho.

[CR33] OECD (2013). The 2012 labour market reform in Spain: a preliminary assessment.

[CR34] Olea Ferreras, S. (2015) *Responses of regional governments in Spain to the Housing emergency and exclusión: The social function of housing*. http://www.housingrightswatch.org/

[CR35] Robertson R, Serpa R, Scanlon W, Fernandez Arrigoitia M (2014). Social housing in Scotland. Social housing in Europe.

[CR42] Ruiz Almendral V (2003). The asymetric distribution of taxation powers in the Spanish state of autonomies: The common system and the foral tax regimes. Regional and Federal Studies.

[CR36] Sollé Ollé, A. (2013) *Regional tax autonomy in Spain: ‘words’ or ‘deeds’*? Workshop KIPF and Danish Ministry of welfare, 12–13 September 2013.

[CR37] Taylor-Gooby P (2004). New risks, new welfare: The transformation of the European welfare state.

[CR38] Tejedor Bielsa J (2012). Derecho a la vivienda y Burbuja inmobiliaria. Editorial La Ley.

[CR39] Vampa, D. (2014) *Territorial mobilization and sub*-*state welfare governance in Italy and Spain. Comparing four regional case studies*. Conference paper.

[CR41] Winters S, Elsinga M (2008). The future of Flemish social Housing. Journal of Housing and the Built Environment.

